# Supplementary material for: A top-down insular cortex circuit crucial for non-nociceptive fear learning
Source: Sci Adv. 2025 May 9;11(19):eadt6996. doi: 10.1126/sciadv.adt6996 (PMC12063665; doi:10.1126/sciadv.adt6996)
Supplement: Supplementary file 1 — Figs. S1 to S6 Legend for table S1 Legend for data file S1 [file sciadv.adt6996_sm.pdf]

Science Advances
AAAS

Supplementary Materials for

A top-down insular cortex circuit crucial for non-nociceptive fear learning

Junho Han *et al.*

Corresponding author: Jin-Hee Han, han.jinhee@kaist.ac.kr

Sci. Adv. **11**, eadt6996 (2025)
DOI: 10.1126/sciadv.adt6996

The PDF file includes:

Figs. S1 to S6
Legend for table S1
Legend for data file S1

Other Supplementary Material for this manuscript includes the following:

Table S1
Data file S1

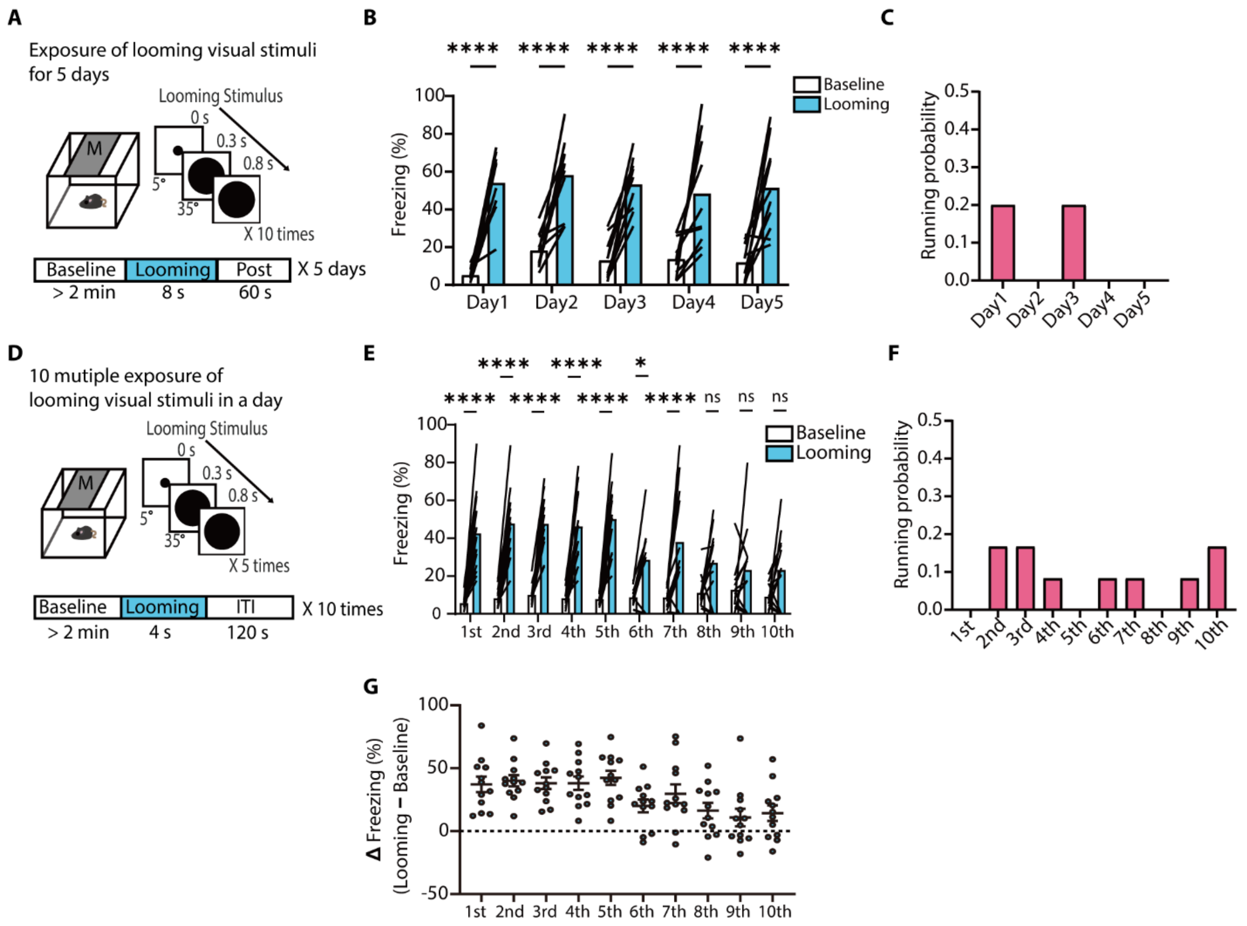

fig. S1. CGRP^{PBN} neuronal activity in response to flickering and looming visual stimuli (related to Figure 1).

(**A**) Schematic of repeated behavioral tests for innate defensive responses to looming visual stimuli over 5 days. Each day, mice were exposed to 10 consecutive looming stimuli (8 s per trial).
(**B**) No significant habituation to daily looming stimuli over 5 days. Freezing responses to looming stimuli remained consistent across days (*N* = 10 mice; RM two-way ANOVA, Stimulus, $F_{1,18}$= 72.07, P< 0.0001).
(**C**) Probability of running responses to daily looming stimuli over 5 days, calculated as the number of mice exhibiting running behavior divided by the total number of mice.
(**D**) Schematic of a behavioral test for habituation of the innate freezing response to looming stimuli. A set of 5 looming stimuli (4 s each) was repeated 10 times with a 120-s intertrial interval.
(**E**) Habituation of innate freezing response. Freezing was measured during each 4-s looming stimuli (*N* = 12 mice; RM two-way ANOVA, Trials × Stimulus, $F_{9,198}$= 4.198, P< 0.0001).
(**F**) Probability of running event to the 10-repeated looming stimuli.
(**G**) Difference in freezing levels between baseline and looming stimuli (RM one-way ANOVA, $F_{9,99}$= 4.598, P< 0.0001).
All data are presented as mean ± s.e.m. *p < 0.05, ****p < 0.0001. ns, not significant.

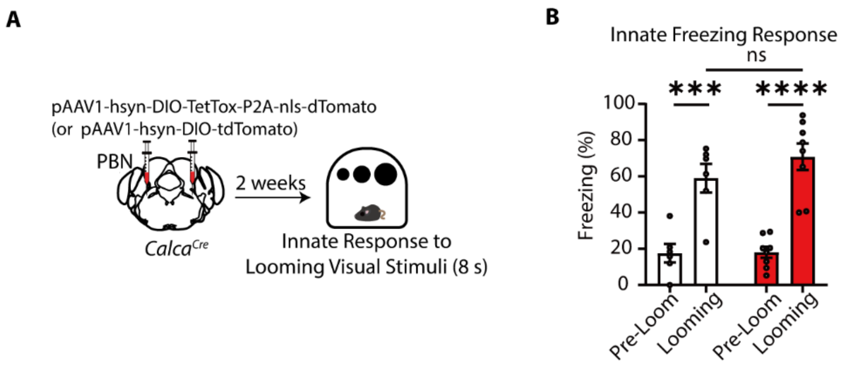

fig. S2. TetTox inhibition of CGRP^{PBN} neurons did not impair innate defensive behavioral responses to looming visual stimuli (related to Figure 3).

(**A**) Schematic of innate freezing response test with TetTox-mediated inhibition of CGRP^{PBN} neurons.
(**B**) TetTox inhibition of the CGRP^{PBN} neurons did not affect innate freezing response to looming visual stimuli (tdTomato group, N= 6 mice; TetTox group, N= 8 mice; RM two-way ANOVA, Time, $F_{1,12}$= 68.01, P< 0.0001).
All data are mean ± s.e.m. ***p < 0.001, ****p < 0.0001. ns, not significant.

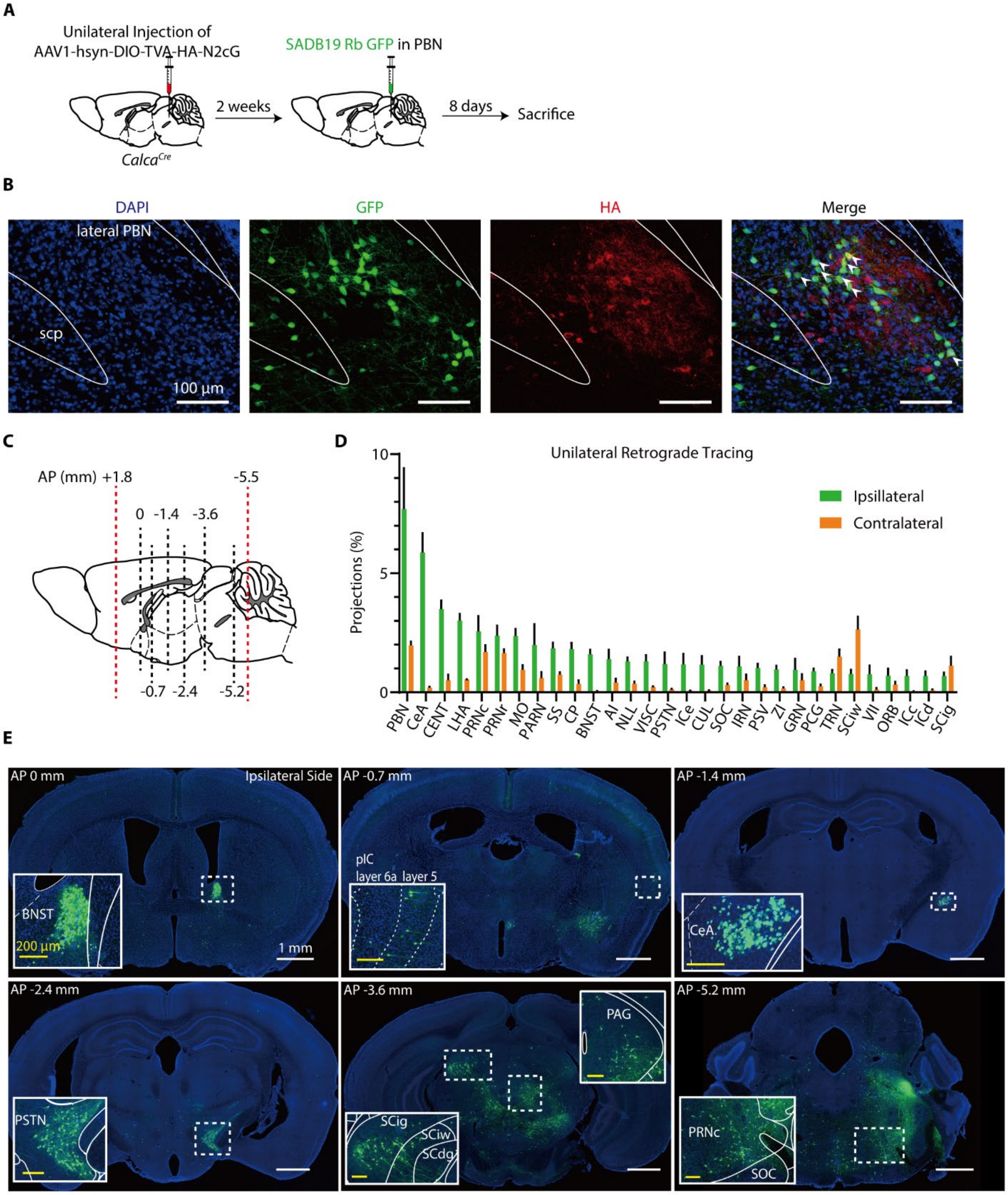
A
Unilateral Injection of
AAV1-hsyn-DIO-TVA-HA-N2cG
SADB19 Rb GFP in PBN
2 weeks
8 days
Sacrifice
Calca^Cre
B
DAPI
GFP
HA
Merge
lateral PBN
scp
100 µm
C
AP (mm) +1.8
-5.5
0 -1.4 -3.6
-0.7 -2.4 -5.2
D
Unilateral Retrograde Tracing
Ipsillateral
Contralateral
Projections (%)
10
5
0
PBN CeA CENT LHA PRNc PRNr MO PARN SS CP BNST AI NLL VISC PSTN ICe CUL SOC IRN PSV ZI GRN PCG TRN SCiw VII ORB ICc ICd SCig
E
AP 0 mm
Ipsilateral Side
BNST
200 µm
1 mm
AP -0.7 mm
pIC
layer 6a layer 5
AP -1.4 mm
CeA
AP -2.4 mm
PSTN
AP -3.6 mm
PAG
SCig
SCiw
SCdg
AP -5.2 mm
PRNc
SOC

fig. S3. Brain-wide monosynaptic retrograde tracing of CGRP^{PBN} neurons (related to Figure 4).

(**A**) Schematic of unilateral monosynaptic retrograde tracing of CGRP^{PBN} neurons. Unilateral injections of AAV2/1-hsyn-DIO-TVA-HA-N2cG into the PBN of *Calca*^{Cre} mice were followed by unilateral injections of SADB19-GFP rabies virus into the same site 2 weeks later.

(**B**) Representative confocal microscopic images of coronal sections showing neurons expressing GFP and/or haemagglutinin (HA) tag in the PBN. White arrows indicate double-labeled starter cells (GFP+ & HA+). Scale bar, 100 µm.

(**C**) AP range of coronal sections collected for imaging analysis (AP, +1.8 mm to -5.5 mm, indicated by dotted red lines) and AP coordinates of representative images shown in (E) (dotted black lines).

(**D**) Distribution of retrogradely labeled neurons across the top 30 brain regions with the highest number of projections.

(**E**) Representative images of coronal sections showing GFP-labeled retrogradely traced neurons in each analyzed target region. Insets show magnified views of the dotted boxes. Scale bars, 1 mm (white bar) and 200 µm (yellow bar in insets).

Abbreviations: Agranular insular area (AI); Bed nuclei of the stria terminalis (BNST); Caudoputamen (CP); Central amygdalar nucleus (CeA); Central lobule (CENT); Culmen (CUL); Facial motor nucleus (VII); Gigantocellular reticular nucleus (GRN); Inferior colliculus, central nucleus (ICc); Inferior colliculus, dorsal nucleus (ICd); Inferior colliculus, external nucleus (ICe); Intermediate reticular nucleus (IRN); Lateral hypothalamic area (LHA); Nucleus of the lateral lemniscus (NLL); Orbital area (ORB); Parabrachial nucleus (PBN); Parvicellular reticular nucleus (PARN); Parasubthalamic nucleus (PSTN); Pontine central gray (PCG); Pontine reticular nucleus, caudal part (PRNc); Pontine reticular nucleus (PRNr); Principal sensory nucleus of the trigeminal (PSV); Somatomotor areas (MO); Somatosensory areas (SS); Superior colliculus, motor related, intermediate gray layer (Scig); Superior colliculus, motor related, intermediate white layer (Sciw); Superior olivary complex (SOC); Tegmental reticular nucleus (TRN); Visceral area (VIS); Zona incerta (ZI).

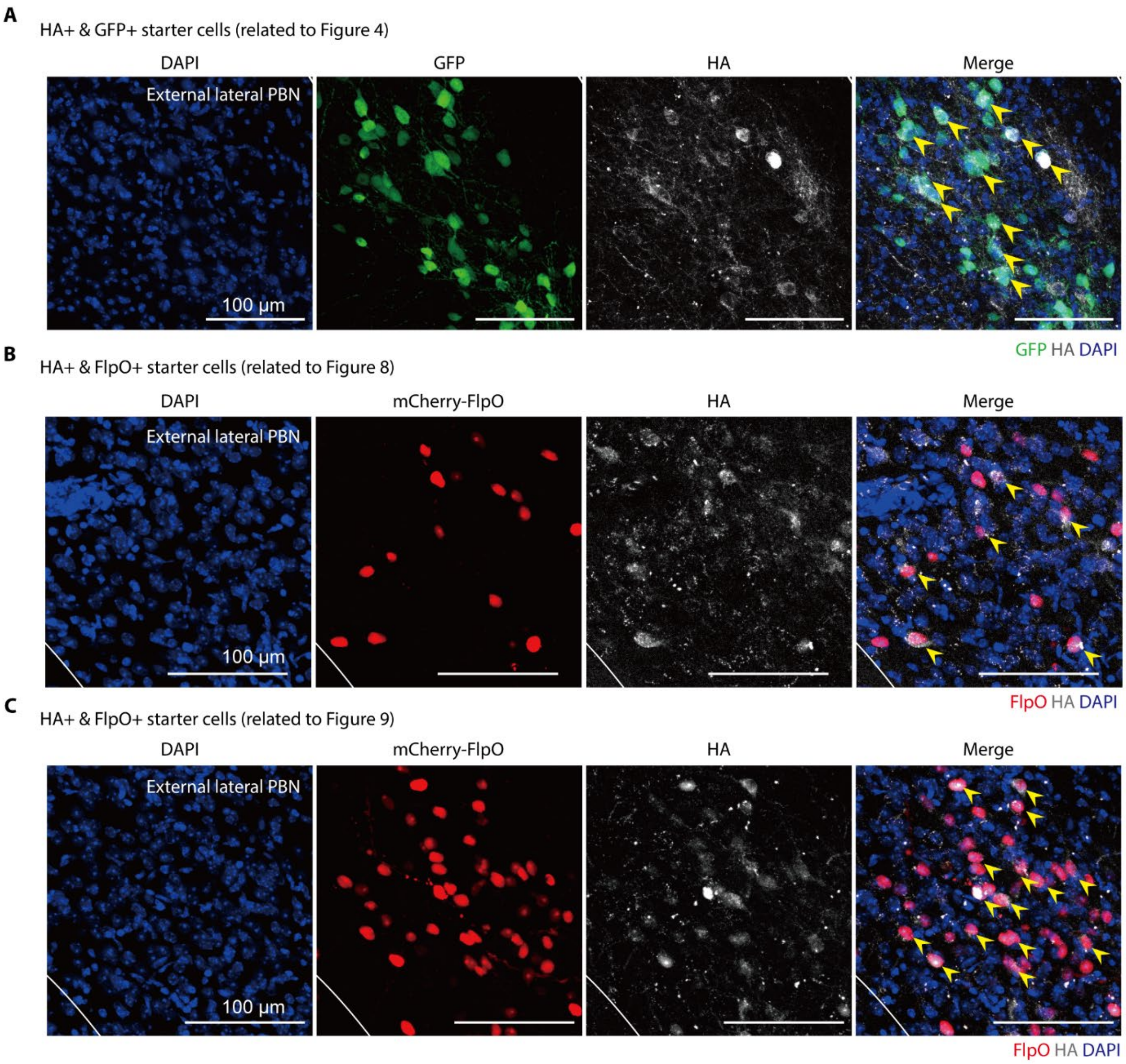

fig. S4. Images of starter cells in the PBN in the monosynaptic retrograde labelling experiments (related to Figure 4, 8, and 9).

(**A**) Representative confocal microscopic images of coronal sections showing the neurons double-labeled for GFP and HA tag in the PBN. Yellow arrowhead indicates double-labeled starter cells (GFP+ & HA+) in Figure 4.

(**B, C**) Representative confocal microscopic images of coronal sections showing the neurons expressing FlpO fused with mCherry and HA tag in the PBN, related to the experiments in Figure 8 (B) and Figure 9 (C). Yellow arrowhead indicates double positive starter cells (mCherry-FlpO+ & HA+). Scale bars, 100 μm.

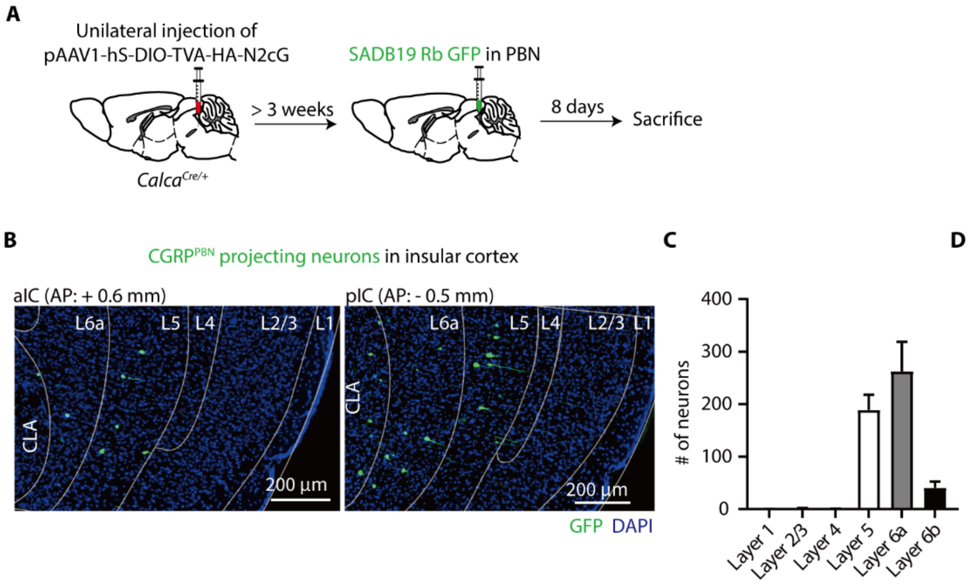

fig. S5. Monosynaptic retrograde tracing of CGPR^{PBN} neurons in the insular cortex (related to Figure 4).

(A) Schematic of unilateral monosynaptic retrograde tracing of CGRP^{PBN} neurons.

(B) Representative images of coronal brain sections showing retrogradely labeled neurons with GFP in the anterior (left) and posterior (right) parts of the insular cortex.

(C) CGRP^{PBN}-projecting posterior insular neurons are specifically located in layers 5 and 6.

(D) Percentage of projections in the anterior and posterior insular cortex (N= 6 mice; Two-tailed paired t-test, t_5= 2.815, P= 0.0373).

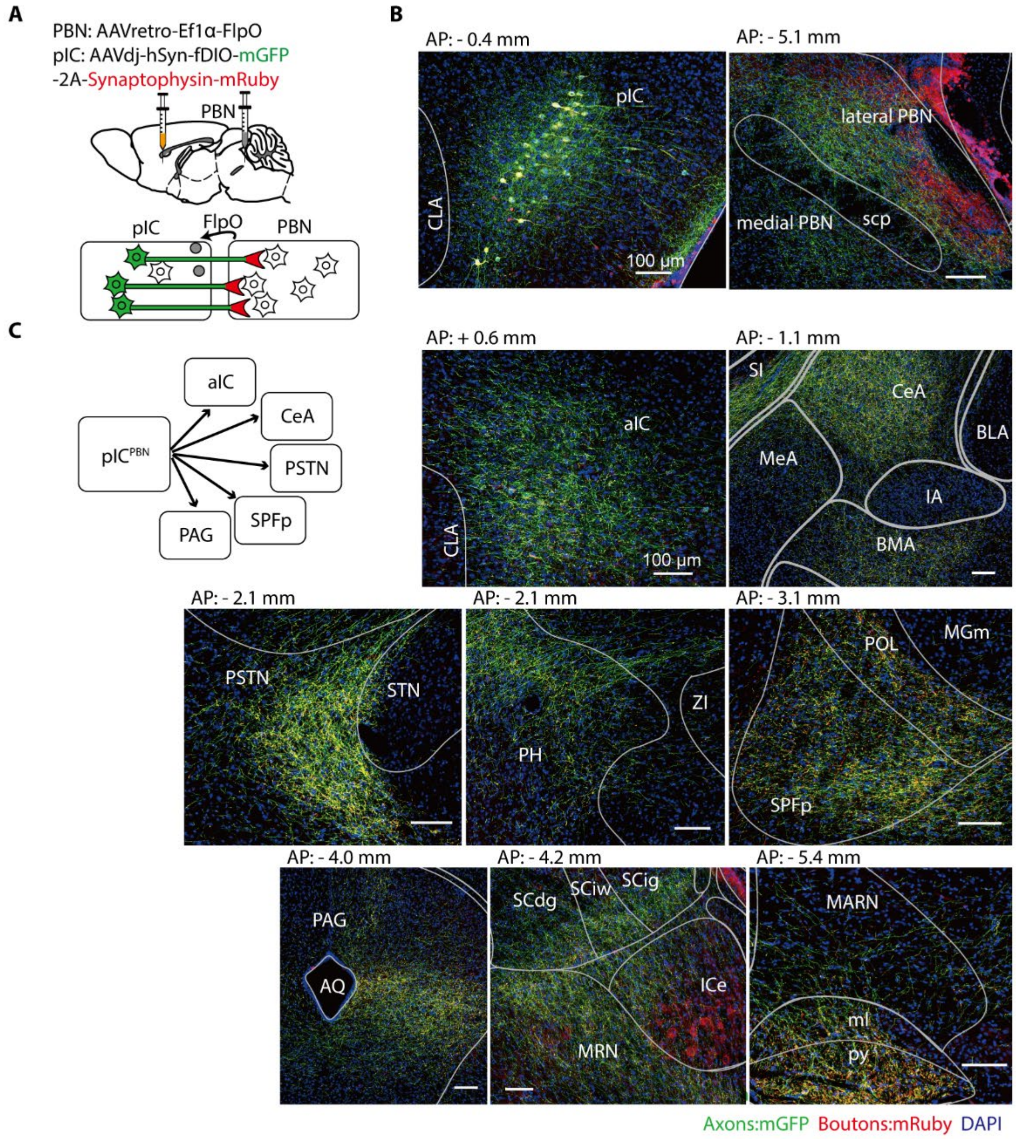

fig. S6. Axon tracing of pIC$^{\rightarrow PBN}$ neurons (related to Figure 6).
(A) Schematic of unilateral axon tracing of pIC$^{\rightarrow PBN}$ neurons. Unilateral injections of AAVretro-EF1α-flpO into the PBN and AAVdj-hSyn-FLEX-mGFP-2A-Synaptophysin-mRuby into the pIC.
(**B**) Representative images of pIC$^{\rightarrow PBN}$ neurons (left) and projections to the PBN (right). Scale bars, 100 µm.
(**C**) Representative confocal microscopic images of coronal sections showing the axons (mGFP+) and synaptic boutons (mRuby+). Scale bars, 100 µm.

Abbreviations: Anterior insular cortex (aIC); Basolateral amygdalar nucleus (BLA); Basomedial amygdalar nucleus (BMA); Central amygdalar nucleus (CeA); Cerebral aqueduct (AQ); Claustrum (CLA); Inferior colliculus, external nucleus (ICe); Intercalated amygdalar nucleus (IA); Magnocellular reticular nucleus (MARN); Medial amygdalar nucleus (MeA); Medial geniculate nucleus, medial part (MGm); Medial lemniscus (ml); Midbrain reticular

nucleus (MRN); Parasubthalamic nucleus (PSTN); Periaqueductal gray (PAG); Posterior hypothalamic nucleus (PH); Posterior insular cortex (pIC); Posterior limiting nucleus of the thalamus (POL); Pyramid (py); Subparafascicular nucleus, parvicellular part (SPFp); Substantia innominata (SI); Subthalamic nucleus (STN); Superior cerebellar peduncle (scp); Superior colliculus, deep gray layer (SCdg); Superior colliculus, intermediate gray layer (SCig); Superior colliculus, intermediate white layer (SCiw); Zona incerta (ZI).

Table S1. (Separate file)
Statistical data for Fig. 1 to Fig. 9 and fig. S1 to fig. S6.

Data file (Separate excel file)
Data sets collected from this study
